# The Influence of Attention and Target Identification on Saccadic Eye Movements Depends on Prior Target Location

**DOI:** 10.1155/2014/850606

**Published:** 2014-02-27

**Authors:** David R. Hardwick, Timothy R. H. Cutmore, Trevor J. Hine

**Affiliations:** Behavioural Basis of Health, Griffith Health Institute, and School of Applied Psychology, Griffith University, Mt Gravatt, QLD 4122, Australia

## Abstract

Saccadic latency is reduced by a temporal gap between fixation point and target, by identification of a target feature, and by movement in a new direction (inhibition of saccadic return, ISR). A simple additive model was compared with a shared resources model that predicts a three-way interaction. Twenty naïve participants made horizontal saccades to targets left and right of fixation in a randomised block design. There was a significant three-way interaction among the factors on saccade latency. This was revealed in a two-way interaction between feature identification and the gap versus no gap factor which was only apparent when the saccade was in the same direction as the previous saccade. No interaction was apparent when the saccade was in the opposite direction. This result supports an attentional inhibitory effect that is present during ISR to a previous location which is only partly released by the facilitative effect of feature identification and gap. Together, anticipatory error data and saccade latency interactions suggest a source of ISR at a higher level of attention, possibly localised in the dorsolateral prefrontal cortex and involving tonic activation.

## 1. Introduction

Saccadic latencies are not simply explained by physiological processes like neural conduction: both task instructions and attention also play important roles [1]. The present study focused on three such factors (among several known to have influence) that have each been shown to reduce saccadic latency: a saccade to a new location that has a shorter latency relative to a saccade that immediately returns to a previously fixated location (known as inhibition of saccadic return, “ISR” [2]), the “gap effect” which is the disappearance of the fixation point before target appearance [3–5], and the need to identify high-resolution detail at the saccadic endpoint [6, 7].

ISR is a specific form of the more general construct of inhibition of return (IOR) which can be applied to many behavioural responses. For example, key-press responses to targets in new locations produce shorter RTs than targets in the same location [8–10]. The ISR effect is thought to contribute to efficiency of visual search [11, 12], such that the observer does not make an unnecessary immediate saccade to previously viewed locations [8, 9, 13, 14]. This is reflected in the neurology: Prime and Ward [15] found reduced amplitude of occipital event related potentials (ERPs) for targets at cued locations when compared to targets at uncued new locations and Van der Lubbe et al. [10] concluded that their ERP, electrooculography (EOG), and button-press RT data excluded a bottom-up premotor response theory of ISR. On the other hand, Hunt and Kingstone [8] proposed that there are both top-down (goal-directed and attention mechanisms) and bottom-up (stimulus-driven) influences on ISR. It should be noted that the shorter latencies due to ISR are not always reported in experimental paradigms. When participants are required to make a series of saccades either towards a target (“prosaccades”) or in the opposite direction to a target (“antisaccades”), saccades were executed more quickly when the saccade in the penultimate trial was in the same direction [16]. This was attributed to low-level phenomenon of “directional plasticity,” where on a given trial it is easier for activity in the superior collicular neurons to reach a threshold to trigger a saccade in the same direction as the prior saccade.

To saccade to a new location, visual attention needs to be released from the previous location and this requires some time [5, 17]. The operation of this process has been revealed in the “gap effect” paradigm. The gap refers to a delay (between 100 and 300 ms) that is introduced between the fixation point offset and the onset of a new target [4]. Gap trials have been shown to markedly reduce saccadic latency and in some studies, a second peak at 120 ms has been noted in the latency distributions in addition to the usual 200 ms peak [17, 18].

Reuter-Lorenz et al. [19] considered the gap effect to be partly due to the cueing component of fixation-offset. They showed that a warning tone by itself prior to fixation-offset in the gap condition, or during fixation in the no gap (temporal “overlap” condition), produced significant reduction in saccade latency. Following this, Pratt et al. [20] cued visual attention to various parts of a fixation cross. When participants were attending to part of the fixation cross that disappeared at the beginning of a gap, they showed significantly reduced latency compared to when they were attending to another part of the cross that did not disappear. Both Hutton [21] and Jin and Reeves [5] concluded that these results support an attentional component in the gap effect. However, there is some dispute as to the neural circuitry underlying this component [22, 23]. Attentional control could be exerted from a number of higher cortical centres to the fixation neurons in the superior colliculus (SC) that generate the saccade. This neural control centre could include the dorsolateral prefrontal cortex (DLPFC) which is involved in saccadic suppression, the frontal eye fields (FEF), and/or the lateral-intraparietal area of the posterior parietal cortex (LIP, [22, 23]).

Trottier and Pratt [7] found that saccadic latency is reduced during discrimination of high-resolution detail at the target location (*identification*), without the gap delay described above. Terminological note: Trottier and Pratt [7] used *look-obtain* (feature of target) versus *look* (without obtaining feature), while others [24, 25] used *identification* versus *glance*. We used *identification* versus *no identification*. Specifically, when naive participants were asked to identify the pixel offset at the centre of a target during fixation overlap trials, saccade latencies were in the very fast range (*M* = 135 ms) and were significantly shorter than no identification fixation overlap trials (*M* = 185 ms). They stated that these very fast latencies could be the norm in naturalistic settings where identification of a new target is the purpose of a saccade and that saccade latencies over 150 ms were in fact abnormally slowed by simplistic laboratory paradigms. Trottier and Pratt [7] proposed that there is increased facilitation producing shorter saccade latency via top-down input to SC from the frontal cortex (including the PFC) during the identification task.

During behavioural studies in which participants were instructed to make pro- and antisaccades (a saccade to an opposite location where the targets appear), Guyader and colleagues [24, 25] replicated Trottier and Pratt's [7] results in showing that removal of gaze fixation object prior to target presentation (the gap paradigm) produced extremely small saccadic latencies when there was a requirement for target identification. However, the effect of identification that was observed during prosaccades (i.e., prosaccade latency was reduced during identification task) was not observed during antisaccades [24]. Guyader and colleagues [24, 25] explained these results as being due to the fact that antisaccades require extra executive processes compared to prosaccades: first, an inhibition of a reflexive saccade to a suddenly appearing target by a signal originating in the prefrontal cortex (PFC), and second, a voluntary saccade to look at the mirror position. They proposed that the identification effect occurs at a high executive level in the saccadic neural pathway but is a different signal from that used in antisaccades. The present study extends the results of these two studies by examining how these attentional factors—gap and identification—interact with contextual ISR, a factor that involves high-level cognitive processes that are sensitive to expectations accumulated over a period of time [2]. In summary, evidence supports the hypothesis that each of the three factors—identification, gap, and ISR—may exert their influence in reducing saccadic eye movement latency through neural activity involving the PFC. However, if these mechanisms converge in a common functional pathway their effects may not be simply additive. In fact, Guyader et al. [24] claimed that they may “cancel” each other. There are studies that have combined two of these three attentional effects, with a reduction in saccade latency greater than in each effect alone [7, 8, 14]. Trottier and Pratt [7] used both a feature identification task and the gap effect and found a significant interaction, such that the reduction in saccadic latency due to correct identification was greater for overlap trials than for gap trials, a result replicated by Guyader et al. [24]. A possible basis for this could be that the gap effect and target identification facilitate saccadic latency via a shared neural pathway from the PFC that disinhibits SC. On the other hand, the identification effect was not present in antisaccades and Guyader et al. [24] surmised that the additional executive processes required for the antisaccades “cancelled out” the effect completely.

It follows that if ISR is the result of an attentional top-down mechanism similar to that required in antisaccades, then it would be expected that Trottier and Pratt's [7] feature identification task, which decreases saccade latency in fixation overlap conditions, might also interact with ISR (cf. results of Terry et al. [26] in motor tasks). For example, on a trial in which the gaze “returns” to the previous location and also requires target identification, ISR may cancel the facilitating effect of the identification process. However, if there is an oculomotor bottom-up component to ISR, then top-down attentional decision tasks may not be affected by this “hard-wired” effect. Therefore, when considered together, the top-down pathway proposed by Van der Lubbe et al. [10] for ISR could have a mediating influence over the top-down pathway proposed for target identification [7, 24, 25] and over the gap effect, producing a three-way interaction. Thus, the strength of the interaction between identification and gap found by Trottier and Pratt [7] would depend on ISR influence. In effect, ISR is implicit in any saccade latency study that randomly presents targets to a few locations that are on either side of a central fixation: about half the trials will be ipsilateral to the previous saccade and half will be contralateral. Trottier and Pratt's [7] results are an average of these trials: the present study makes this laterality an independent variable to determine the ISR effect.

If they are wholly independent, then combining all the factors would result in simple additive main effects with the possibility of at least one of these (e.g., the gap effect) being due to bottom-up control mechanisms. Less than additive effects, that is, some type of two- or three-way interaction, would support the notion that a shared resource is being used and this could help to circumscribe how these attentional factors might be linked to the underlying neural mechanisms. While it is clear that behavioural data alone are insufficient to identify a brain source associated with a limited shared resource, such data may be used to suggest possible places to examine with electrophysiological recording methods. Certainly, Malsert et al. [25] theory allows for sharing of such top-down control resources, but only for what they describe as “tonic” PFC activation—a slow change in neural response due to identification as opposed to “phasic” PFC activation which only lasts for fractions of a second at the onset of a task.

Finally, anticipatory errors occur when participants move their eyes prior to appearance of the target (either during fixation or during the gap) or move their eyes after appearance of the target but before they could possibly have processed the target. Such errors have been assumed to be an important indicator of disinhibition of top-down control of SC circuits [27]. For example, Trottier and Pratt [7] reported a greater anticipatory error rate during identification and gap trials compared to other trials and assumed this disinhibition increased during target feature identification resulting in a faster subsequent saccade. Attentional disengagement is also facilitated by both the gap effect and by there being an opposite location (contralateral) to the previous target [8, 14, 28], that is, in the presence of an ISR effect. This disengagement toward new locations is partly mediated through the SC, permitting it to more freely initiate an eye movement, even in the absence of a target as occurs in antisaccades [24] and particularly if the frontal eye field (FEF) is contributing to anticipatory errors [29].

Bearing this in mind, one contribution of the present study is that it predicts that conditions facilitating this disengagement (e.g., during identification versus no identification) will result in more anticipatory saccadic errors, but particularly when the previous target appeared in a contralateral location to the current target, the conditions of ISR. Further, depending on whether the previous target is contralateral or ipsilateral to the current target, significant differences are also predicted for the type of anticipatory saccades. For contralateral prior location during identification trials, anticipations towards the target (protarget anticipations) would be greater than anticipations away from the target (antitarget anticipations). Furthermore, for ipsilateral prior location, during identification trials, antitarget anticipations would be greater than protarget anticipations. However, we propose that when PFC disinhibition of saccades does not identify a target feature (during no identification trials), protarget and direction anticipation rates will be approximately equal for contralateral and ipsilateral target prior location.

## 2. Materials and Method

### 2.1. Participants

Twenty naïve first-year psychology students with normal vision, 11 females and 9 males (age *M* = 22 years, SD = 6, and range = 17 to 35 years), participated in the study in exchange for course credit. Six other participants did not complete the trials.

### 2.2. Design

**Figure 1:**
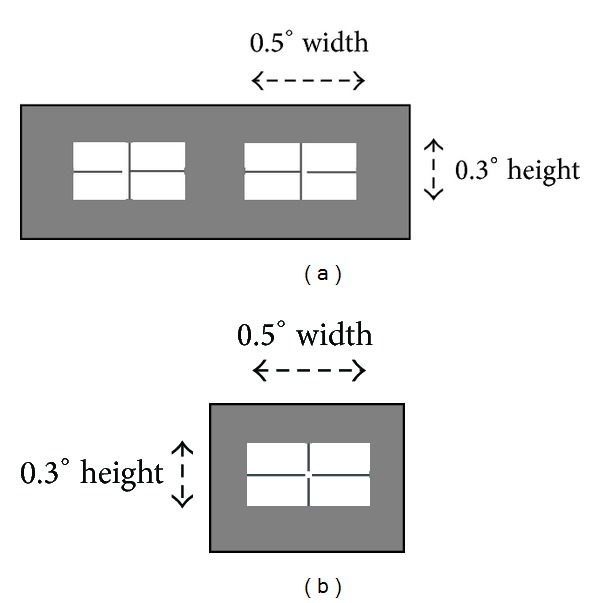
Saccadic stimuli for identification of fine features (after [7]). For a *feature identification* task, one of two stimuli (a) was presented with a single pixel offset either left or right, either target being presented randomly left or right of central fixation. In a *no identification *task, stimulus on the right (b) with a central pixel was presented in the same manner.

The main experiment was a five-way “gap” × “identification” × “target prior location” × “direction” × “eccentricity” repeated measures factorial design. There were two levels each of temporal “gap” (200 ms gap/no gap), “identification” (identification/no identification), “target prior location” (ipsilateral/contralateral), and “direction” (left/right) and three levels of “eccentricity” (4°, 6°, or 8°), giving 48 within-subject data cells. Saccadic latency, the percent of anticipatory saccades, and key-press reaction time (RT) from target onset were all measured, as was accuracy in the pixel offset discrimination task (Figure 1). Both gap levels and identification levels were blocked.

### 2.3. Apparatus

Each eye was sampled at 1000 Hz with an IRIS infrared eye tracker [30, 31] with spatial accuracy <0.25°. Head position was stabilised at 57 cm viewing distance with a custom-built chin and cheek rest. Stimuli were displayed on a 21-inch CRT monitor running at 85 Hz horizontal refresh and 1600 × 1200 pixel resolution and were aligned horizontally half-way down the screen. Stimulus luminance was approximately 17.5 cd/m^2^. The display background luminance was adjusted upward until the phosphor decay was not visibly noticeable (a little less than 0.20 cd/m^2^). The start of the raster at the top left of the screen was synchronised with the eye trace record and corrected offline for scanning delay (5 ms). A PC running Neurobehavioural Systems Presentation program was used to display and collect data.

The infrared eye tracker centre position was adjusted for each participant prior to stimulus calibration. There were nine calibration trials at the beginning of each trial block, followed by six practice trials. Calibration targets with pseudorandom positions had the dimensions depicted in Figure 1(b) and were shown across 9 locations ranging from –10° to +10°, in 2.5° intervals. Each calibration target remained on for 2000 ms, with a 1000 ms interval between each target. Due to the controlled head movement and shortness of the trial blocks, no further calibrations were necessary.

### 2.4. Stimulus Parameters

Identification was operationalised as discrimination of target centre pixel lateral displacement similar to Trottier and Pratt ([7]; Figure 1). The central fixation crosshair was 0.8° × 0.8° and 0.05° line width.

**Figure 2:**
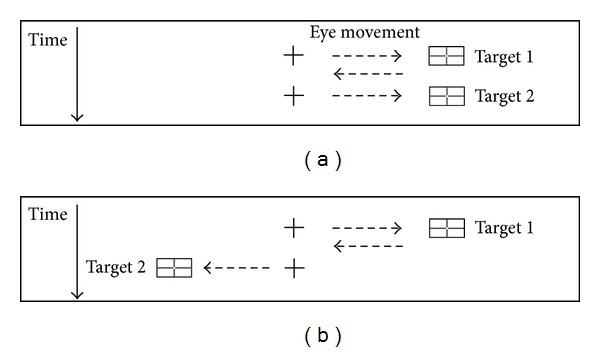
Operationalisation of target prior location for inhibition of saccadic return. Centre cross is fixation. Dashed arrows represent eye movements, with time down the page. (a) is ipsilateral prior location and (b) is contralateral prior location.

The sequence of saccades for *contralateral prior location* and *ipsilateral prior location* (Figure 2) was operationalised in a similar manner to Carpenter [14]. Each second target in a sequence pair became a first target for the next sequence pair, so in a block of 120 trials there were 119 possible pairs.

In general, increased randomisation results in reduced predictability, thus producing higher probability of very fast exogenous reflexive saccades [32]. Therefore, consistent with [8, 33], in order to increase uncertainty and ensure fast exogenous saccade production, targets were both temporally and spatially randomised. Accordingly, right and left direction from fixation, target eccentricity, duration of intertrial interval, and duration of fixation cross were all continuously randomised. Multiple locations were presented along the horizontal plane in randomised order: ±4°, ±6°, and ±8° eccentricities.

### 2.5. Procedure

There were four Latin square counterbalanced trial blocks, each with 120 trials: no identification, no identification-gap, identification, and identification-gap. If the no identification and identification trials had been interleaved this would have introduced a third choice (press left, press space bar, or press right), without any control task. Separating them into blocks largely reduced this effect, although there was probably a short-lasting carryover effect of identification when the counterbalanced order called for the identification block to occur first. The only way to control this carryover, apart from counterbalancing, would be to conduct a much larger between-subject study. These were conducted in one session in the laboratory. The ISR factor was derived post hoc. For identification trials (Figure 1(a)), participants were instructed to “immediately look at the centre of the peripheral target and identify the direction in which the central pixel is offset” and then “press the appropriate arrow key, left arrow for left offset and right arrow for right offset.” For no identification trials (Figure 1(b)), participants were instructed to “immediately look at the centre of the peripheral target” and then “immediately press the space bar.”

Response instructions were displayed on the screen prior to the calibration, prior to the practice trials, and prior to the main trials for each block of trials. Each participant was instructed to keep their head as still as possible on the chin-rest during testing. Participants were instructed that accuracy and speed were equally important across all conditions for both eye movement and key-press response.

Each trial started with a white crosshair at central fixation. Both fixation duration and intertrial interval continuously randomly varied between 500 and 1000 ms. A new intertrial interval, which also determined the beginning of the interstimulus interval, began once a response key was pressed. These values were greater by 200 ms in gap trials due to the delay in stimulus onset. If a key-press response was not made within 1500 ms of target presentation the trial timed out and the message “Press key to continue” appeared. Note that the interstimulus interval for presentation of the target also defined the intersaccade interval for ISR and was well within the range for the ISR effect to occur, which has been estimated to be between 500 and 3500 ms [9].

Anticipatory saccades were identified offline. An accepted method of finding within-subject criteria for anticipations is to determine the particular saccade latency at which direction errors cease to occur for each participant [7, 32]. Hence, saccades made opposite to target direction were counted as anticipations, as were saccades that were made toward the target within the same unacceptably short latency range. Terminal position inaccuracy and double saccades are also indicative of anticipatory saccades. For example, when an initial saccade terminal position was far from the target (defined here as horizontally greater than 1.5°), it was usually followed by a corrective saccade within 50 to 60 ms; the initial saccade was therefore coded as anticipatory. Moreover, trials on which anticipatory key-presses occurred were excluded automatically during testing, as each trial was terminated by a key-press.

## 3. Results

Because it was prerequisite that participants have normal vision, the eye trace data for each eye was compared for each participant to ensure that there were no anomalies. After choosing the eye with the cleanest signal the vertical-axis voltage signal was calibrated to obtain approximate position in degrees. This normally resulted in the right eye trace being analysed. Saccadic latency onset after target presentation was automatically determined using a Matlab © routine as that time at which eye velocity was approximately 20°/s. These values were scanned for anomalies, and in very rare cases saccade onset was manually coded or the trial coded as an error. Data were obtained for all legitimate trials: saccadic latency, anticipations, landing position inaccuracy, and blinks. Anticipations and other exclusions were further analysed. Exclusion of saccadic latencies as slow outliers was based on being beyond 3.29 *z*-scores above the mean. Only 0.5% (51/9600) saccadic latencies met the slow outlier criterion. Although the saccadic latency threshold for anticipations varied among participants and among conditions, it was found to occur between 65 and 85 ms. Anticipatory key-press prior to target presentation (0.2%) and blinks (1.7%) appeared to be randomly distributed across conditions.

### 3.1. Saccadic Latency

**Figure 3:**
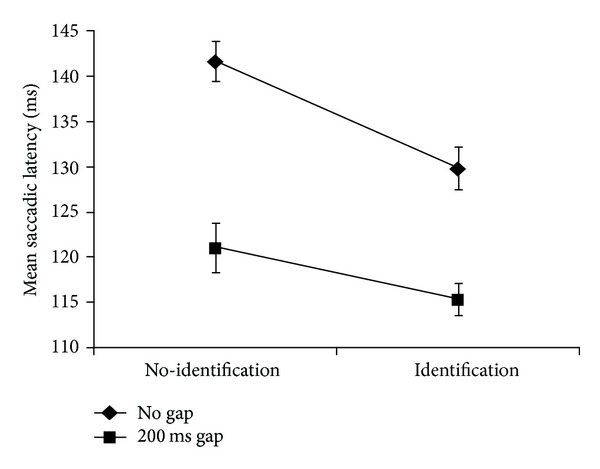
Significant two-way interaction of gap × identification of target feature for saccadic latency. Error bars = ±1 SEM.

**Figure 4:**
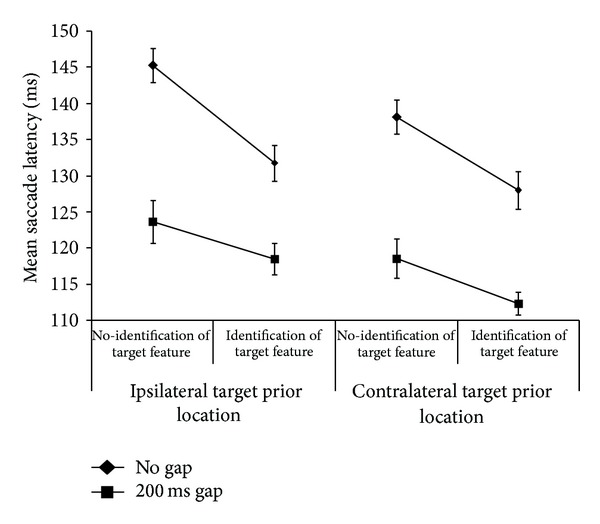
Mean saccadic latencies for *n* = 20 collapsed across eccentricities. Main effects and interactions are described in the text: note the three-way interaction for gap × identification × target prior location. Error bars = ±1 SEM.

A five-way repeated measures factorial ANOVA for the main experimental design was conducted with gap (2) × identification (2) × prior location (2) × direction from fixation (2) × target eccentricity (3), on saccadic latency. Main effects for the following conditions were significant: 200 ms gap < no gap, *F*(1,19) = 87.46, *MSE* = 843.71, *P* < .001; identification < no identification, *F*(1,19) = 24.92, *MSE* = 740.48, *P* < .001, contralateral < ipsilateral *F*(1,19) = 26.83, *MSE* = 275.05, *P* < .001, and target eccentricity, linear effect *F*(1.5, 28.39) = 23.34, *MSE* = 186.03,  *P* < .001 (Greenhouse-Geisser corrected), and target eccentricity quadratic *F*(1,19) = 7.73, *P* = .012, *MSE* = 64.24. The two-way interaction of gap × identification was significant, *F*(1,19) = 10.31, *MSE* = 220.47, *P* = .005 and was similar to previous results ([7], their Figure 2; [24] their Figure 3) and is shown in our Figure 3. These two factors were also involved in a significant three-way interaction of gap × identification × prior location, *F*(1,19) = 5.13, *MSE* = 58.32, *P* = .035, which is shown in Figure 4.

This three-way interaction of gap × identification × prior location was further analysed by a two-way repeated measures ANOVA for gap × identification for each of the two ISR levels. There was a significant two-way interaction of gap × identification for ipsilateral targets, *F*(1,19) = 15.98, *MSE* = 132.03, *P* = .001, but not for contralateral targets, *F*(1,19) = 3.33, *MSE* = 63.44, *P* = .084. The left (ipsilateral) panel of Figure 4 shows this two-way interaction. Bonferroni corrected paired comparisons, for the pairs of conditions connected by a line in the graph, showed that identification was significantly faster than no identification for all but the ipsilateral 200 ms gap condition (mean difference = 5.2, SEM = 2.7 ms,  *P* = .067).

**Figure 5:**
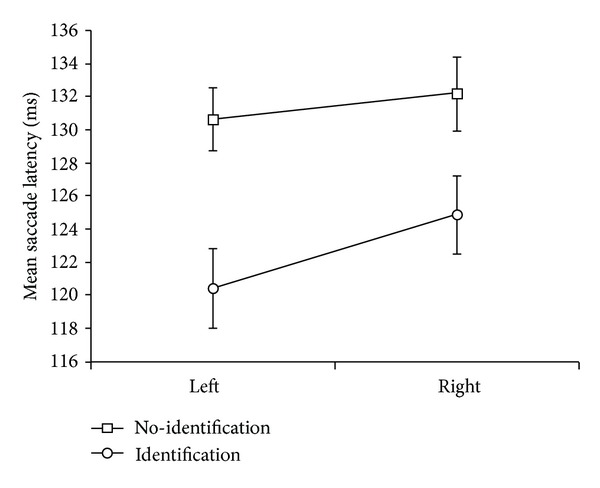
Mean saccadic latencies for the two-way interaction of identification × direction from fixation. Error bars = ±1 SEM.

**Figure 6:**
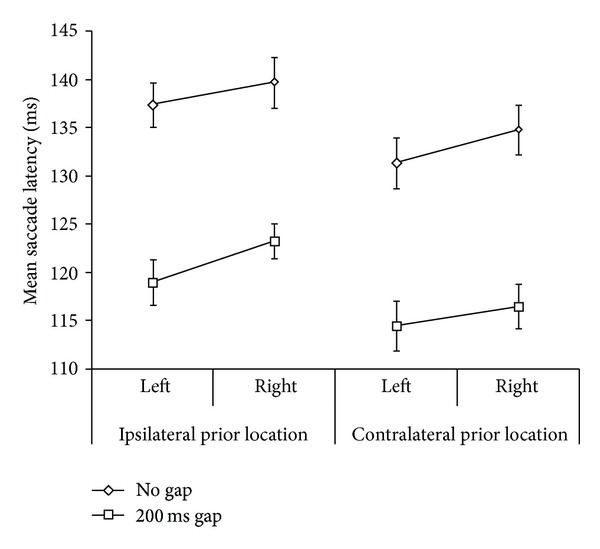
Mean saccadic latencies for the three-way interaction of gap × prior location × direction from fixation. Error bars = ±1 SEM.

The two-way interaction of identification × direction from fixation was significant, *F*(1,19) = 5.68, *P* = .028, *MSE* = 88.43 (Figure 5). Right direction from fixation was significantly greater than left under identification (mean difference = 4.5 ms,  *P* = .016), but not under no identification. The three-way interaction of gap × prior location × direction from fixation was significant, *F*(1,19) = 5.01, *P* = .037, *MSE* = 34.97 (Figure 6). When testing the simple effects of direction from fixation, the only significant post hoc paired comparison was for 200 ms gap under ipsilateral prior location, with left being significantly quicker than right (mean diff. = 4.3 ms, *P* = .049). This comparison is represented by the lower line in the left panel of Figure 6. The other three pairs were not significant.

### 3.2. Anticipation

Anticipatory saccades had low frequencies in no gap condition levels, with no anticipations for some participants, and therefore the comparison of no gap with 200 ms gap was initially explored with Wilcoxon-signed-ranks test. This test found that there was a significantly greater probability of anticipatory saccades for gap trials compared to no gap trials, *z* = 3.92, *P* < .001 (two-tailed). No gap trials median was 3.6% (min. = 0.18%, max. = 7.5%) and 200 ms gap trails median was 8.0% (min. = 2.6%, max. = 17.0%) anticipatory errors.

**Figure 7:**
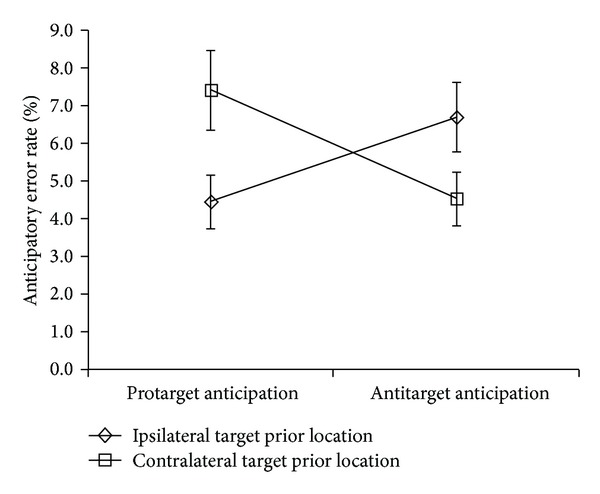
Mean percent of anticipatory eye movements under ISR conditions. Anticipatory eye movement two-way interaction for target prior location × anticipatory type. Note: Protarget = anticipation in same direction as target. Antitarget = anticipation in opposite direction from target. Ipsilateral = same direction target prior location. Contralateral = opposite direction target prior location. Error bars = ±1 SEM.

A repeated measures ANOVA was performed on percent of anticipatory saccades collapsed across gap levels. There was a significant two-way interaction of target prior location × anticipatory type (protarget versus antitarget anticipations), *F*(1,19) = 9.79, *MSE* = 53.45, *P* = .006. Examination of Figure 7 reveals a crossover interaction between target prior location and anticipatory type. Hence, protarget anticipations were more likely when the previous target was in the contralateral location to the current target, whereas antitarget anticipations were significantly more likely when the previous target was in the ipsilateral location.

**Figure 8:**
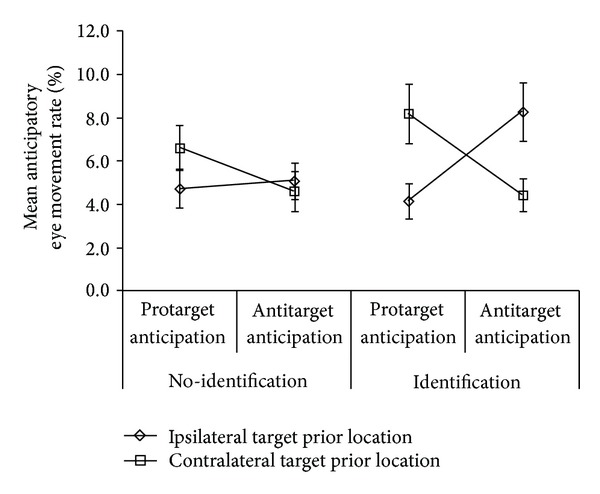
Mean frequency of anticipatory eye movements under ISR conditions. Anticipatory eye movement three-way interaction for identification × target prior location × anticipatory type for 200 ms gap trials. Error bars = ±1 SEM.

There was a significant three-way interaction of identification × target prior location × anticipatory type, *F*(1,19) = 5.48, *MSE* = 52.59, *P* = .030.  Figure 8 shows mean percent anticipation rates. Two-way repeated measures ANOVA across identification levels showed a significant interaction for target prior location × anticipation-type under identification, *F*(1,19) = 7.40, *MSE* = 102.19, *P* = .014, but not under no identification. Examination of Figure 8 reveals a crossover interaction between target prior location and anticipatory type for identification (right panel) which is not present in the no identification condition. Hence, under the identification condition, protarget anticipations were more likely when the previous target was in the contralateral location to the current target, whereas antitarget anticipations were significantly more likely when the previous target was in the ipsilateral location.

### 3.3. Feature-Discrimination Accuracy during Identification

Incorrect key-press for feature discrimination between left and right centre pixel offset comprised 2.8% of trials (93/3200), with relatively even distribution across gap and target prior location condition levels.

### 3.4. Key-Press RT

Repeated measures factorial ANOVA was conducted on the key-press RT data using the same factors as the saccadic latency data. Unsurprisingly, key-press RT for identification requiring discrimination was significantly longer than for no identification trials, *F*(1,19) = 216.82, *MSE* = 78556.55, *P* < .001 (mean difference = 266 ms). Key-press RT for target eccentricity was significant, *F*(1.48,28.15) = 52.32, *MSE* = 4261.70, *P* < .001 (Greenhouse-Geisser corrected); target eccentricity quadratic was also significant, *F*(1,19) = 4.76, *MSE* = 1314.08, *P* = .042. However, key-press RT for target eccentricity was involved in a significant two-way interaction with identification, *F*(1,19) = 5.94, *MSE* = 1995.93, *P* = .025 and quadratic *F*(1,19) = 4.50, *MSE* = 884.86, *P* = .047.  Table 1 shows mean key-press RT for this interaction. There was a significant curvilinear effect of eccentricity under identification, but not under no identification for key-press RT, as shown in the difference scores (Table 2).

There was a significant two-way interaction of identification × target prior location, *F*(1,19) = 5.63, *MSE* = 2063.55, *P* = .028. This was due to key-press RT for no identification being longer for ipsilateral prior location (*M* = 566 ms, SEM = 25) than contralateral (*M* = 559 ms, SEM = 24), whereas key-press RT for identification was shorter for ipsilateral prior location (*M* = 825 ms, SEM = 17) than contralateral (*M* = 833 ms, SEM = 18). That is, the saccadic-IOR (ISR) effect for contralateral locations reducing latencies was *reversed* for manual RT only under the identification discrimination task.

There was a significant two-way interaction of identification × direction from fixation, *F*(1,19) = 5.72, *MSE* = 1900.18, *P* = .027 (see Table 2). Key-press RT to the right of fixation was quicker under no identification, while key-press RT to the left was quicker under identification.

## 4. Discussion

The primary purpose of this study was to measure the interaction of three factors associated with saccadic latency: a temporal gap between the fixation and the target, identification of target feature, and ISR. It was hypothesized that an inhibitory influence by the PFC due to ISR could account for the less than additive effects [7] of gap and identification factors. Overall, results support this hypothesis as revealed in nonadditivity of the individual factors when ISR was present but not when it was absent (i.e., a three-way interaction was observed, Figure 4). Thus, while the individual factors can produce additive shortening of saccade latency, ipsilateral trials must be considered as having a moderating inhibitory (i.e., ISR) effect on the shortening of 200 ms-gap-identification trials. A simple explanation in terms of a floor effect in latencies of the saccadic oculomotor system is unlikely since the contralateral 200 ms-gap-identification saccadic latency is significantly quicker (a very short mean of approximately 112 msec) than the ipsilateral comparison ISR condition (see Figure 4).

### 4.1. Saccadic Latency Interactions

It can be noted that the two-way interaction of gap × identification replicates the results of both Trottier and Pratt [7] and Guyader et al. [24]. In the present experiment, we separated the trials on this additional ISR factor and demonstrated that the two-way interaction only manifests itself when ISR is present, producing a three-way interaction—compare the left (ISR present) and right (no ISR) sections of Figure 4. Trottier and Pratt [7] implicate a top-down mechanism (identification) as responsible for reduced saccadic latency when a target feature is to be identified; however they do not pursue explanation of the two-way interaction. There appears to be a unique effect occurring in the ipsilateral 200 ms-gap-identification condition such that the facilitating top-down effect of instruction-type to identify a target feature is reduced. This functional effect could be due to reduced efficacy in PFC saccadic disinhibition of SC, a mechanism proposed by Trottier and Pratt [7] to account for shorter saccadic latency when the task requires fine target detail identification. With the visual attention disengaged due to the 200 ms gap, when a target appears in a previously fixated location this disinhibition fails to exert the degree of influence it does if the target appears in the ipsilateral location.

A common mechanism for gap and ISR in the frontal cortex, possibly the FEF, could be the basis of this interaction. In fact, an FEF stimulation study conducted with monkeys by Opris et al. [34] concluded that the gap effect may lower the FEF threshold required for initiating a saccade. On the other hand, the locus of the interaction may well be in “tonic” activation of the PFC [25]. According to these authors, tonic activation is required to maintain identification in working memory when saccadic tasks are blocked into instruction types as they are in the current study (2 (identification) × 2 (gap) = four blocks). Only phasic activation is required when there is no such memory requirement and the saccadic task is cued within each trial in a completely mixed trial paradigm. Only tonic activation admits of resource sharing and interaction. Following this line of argument, in the Malsert et al. [25] study, the interaction between antisaccade/prosaccade and identification did not occur in their mixed trial paradigm, whereas in our blocked trial paradigm the interaction between ISR, gap, and identification did occur. Therefore, one possible explanation of the three-way interaction is as follows: when a gap-trial and identification instruction require the participant to identify a target feature, a phasic PFC mechanism common to the gap effect and ISR is activated. The inhibitory ISR influence dominates at this locus negating the facilitating influence in general agreement with [10]'s top-down pathway.

### 4.2. Anticipatory Saccadic Errors

Gap trials produced significantly more anticipatory saccades than the no gap trials. This finding supports the theory that there is a greater state of SC disinhibition in preparation for a saccade during the gap, as found in direct neuronal recording from monkey SC during gap tasks [35, 36]. According to Fischer and Weber [17], freeing fixated attention during the gap releases voluntary control of bottom-up processes and allows initiation of search for a target, which can produce a higher rate of anticipatory saccades.

There was also a greater probability of anticipations during the identification trials as predicted. However, this was subsumed in a three-way interaction of anticipatory type (protarget versus antitarget anticipation), target prior location, and identification. Anticipatory errors were significantly more probable in the direction that the oculomotor system favours, that is, away from the previous target being the expected response (the crossover interaction, right side of Figure 8) that only occurs during the identification condition. The two-way interaction under identification is occurring before the targets can be adequately processed because anticipatory errors by definition occur at a very early stage. This anticipatory saccade three-way interaction may have its basis in an interaction of top-down and bottom-up processes. One possible explanation is that the top-down endogenous effect of target identification during overt discriminatory planning disinhibits reflexive motor mapping and spatial planning, and in this case the effect appeared to allow these areas to trigger anticipatory saccades to *expected* locations in the absence of top-down inhibition from DLPFC and FEF. The anticipatory effect found here is consistent with the oculomotor corollary of Pierrot-Deseilligny et al. [29] finding of increased anticipatory saccades in patients with recently damaged DLPFC. However, this needs to be verified by direct neurophysiological investigation, for example, in monkeys.

Together, the saccadic latency and anticipatory saccade results appear to support a theory of ISR that may be low-level reflexive but certainly involves an expectancy effect that manifests as a shared top-down disinhibitory control during identification. This could allow FEF/supplementary eye fields spatial planning areas to have an anticipatory IOR effect [37]. Further, top-down disinhibition from DLPFC could allow SC saccadic mapping [38] or FEF spatial planning neurons to inadvertently anticipate a target, thus causing an involuntary anticipatory error (e.g., [10, 29]).

The accuracy rate for identification was higher than that found by Trottier and Pratt [7]. They found accuracy of 80% to 90%, whereas in this study pixel offset discrimination accuracy was 97%. The randomisation of multiple aspects of the experimental design most likely contributed to this improvement in accuracy. That is, making the task more unpredictable encouraged participants to respond more carefully (due to increased uncertainty). Furthermore, if being able to predict what was going to happen next had been a factor in causing anticipations, then this would have showed an increased anticipation rate across all types of trials. This was not the case; trials without 200 ms gap and without identification of target feature had very low to zero anticipation rates for some participants.

Moreover, changing temporal and spatial certainty of the upcoming target might change some of the other exogenous effects observed in this study. In particular, anticipatory saccades would likely decline and the three-way interaction for anticipatory error type × prior location × identification could change under more predictable conditions. For example, overlap condition levels were not included in this study. Hunt and Kingstone [8] found an increased ISR effect during fixation overlap tasks compared to no gap tasks. Further, Weber at al. [32] found an increased probability of very fast saccades during randomisation of fixation fore-period (i.e., intertrial interval) for fixation overlap trials. Taken together, these findings suggest that the inclusion of fixation overlap could magnify the interaction effect with gap, identification, and ISR. Also note that while Weber at al. [32] found decreased express saccades when the fixation duration was randomly varied, they used durations of one, two, or three seconds; we used a random variation between 500 and 1000 ms. Hence, our manipulation may not have had the same effect as that of Weber et al. [32].

The anticipatory error data appeared to be particularly sensitive to changes in top-down inhibitory control [27]. It has previously been hypothesised that executive frontal inhibitory deficits are associated with eye movement dysfunction in people diagnosed with schizophrenia, and increased anticipatory saccade rates in the antisaccade task have been used to measure eye movement dysfunction [39, 40]. Our task is far easier to participate in compared to the antisaccade task, which is an important factor for people who are psychologically distressed. Further exploration of our anticipatory error data interaction could see the development of a new tool to compare schizophrenia participants with controls especially as it relates to different levels of tonic PFC activation, and this would add to work already published on pro- and antisaccade latencies [16]. Finally, higher-order interactions between response systems may also occur. The present experiment may be considered to be a dual task in the sense that both saccadic and manual responses are being executed. While it was beyond the scope of this study to investigate possible higher-order “cross-talk,” Huestegge and Koch [41] have shown that such cross-talk may occur under certain conditions. In essence, our data for the three-way interaction of gap × identification × target prior location incorporates cross-talk, but from a different perspective, because it includes (by default during identification trials) analysis of the participant pressing a right or left arrow key on the keyboard, immediately after looking at the target which can also be to the right or the left of fixation.

## Figures and Tables

**Table 1:** Mean key-press RT (KPRT) in milliseconds (ms) for the two-way interaction of target-feature-identification × eccentricity with standard errors (SEM). Columns to right show Bonferroni corrected mean differences for paired comparisons of 4° to 6° and 6° to 8°.

	Eccentricity	KPRT (ms)	SEM	Mean diff.	SEM
No-identification	Four	544	23		
	Six	563	25	19*	4
	Eight	580	25	17*	4

Identification	Four	799	18		
	Six	835	18	37*	5
	Eight	852	17	17*	5

*Paired comparison significant at Bonferroni corrected *P* < .05.

**Table 2:** Key-press RT in milliseconds (ms) for the two-way interaction of target-feature-identification × direction from fixation.

	Direction	KPRT (ms)	SEM	Mean diff.
No-identification	Left	564	24	
	Right	560	25	+4.1

Identification	Left	824	18	
	Right	834	17	−9.4
